# Chemical Composition of Essential Oils and Their Potential Applications in Postharvest Storage of Cereal Grains

**DOI:** 10.3390/molecules30030683

**Published:** 2025-02-04

**Authors:** Jianmei Yu

**Affiliations:** Department of Family and Consumer Science, North Carolina Agricultural and Technical State University, Greensboro, NC 27411, USA; jyu@ncat.edu; Tel.: +1-336-285-4861

**Keywords:** grain storage loss, insect infestation, microbial contamination, essential oils, chemical compositions of essential oils, insecticidal activity, antifungal activity, antibacterial activity

## Abstract

Insect infestation and microbial, particularly mold contamination, are the major causes of stored grain deterioration during postharvest storage, which results in a significant loss in grain quality and quantity, and the formation of toxic chemicals such as mycotoxins. Pesticides, together with physical protection strategies, have been widely used to control insects and molds in stored grains, but their uses present significant environmental and health problems. This has led to the exploration of safer pesticide alternatives. Essential oils (EOs) are highly concentrated materials extracted from leaves, stems, flowers, seeds, roots, fruit rinds, resins, or barks. They are multifunctional due to their complex chemical composition. Thus, EOs are frequently used for their therapeutic, antimicrobial, odoriferous, and flavor properties in a wide range of products like medicine, cosmetics, and foods. This review provides comprehensive information on the chemical compositions of EOs commonly used in the food industry, factors influencing EO composition, and recent studies on the potential of EOs as alternatives to synthetic pesticides and fungicides for stored grain protection. The relationship between chemical compositions of EOs and their anti-insects and antimicrobial potentials, as well as current approaches/technologies of using EOs for food preservation, are also covered. However, this review also highlights the need for research on the development of feasible and affordable methodologies to apply effective EOs or encapsulated EOs in grain storage settings, particularly for organic grain protection.

## 1. Introduction

### 1.1. Significance of Postharvest Loss of Crops

Grains, including cereal grains (corn, rice, wheat, oat, and barley) and legumes, are important crops for human and animal foods. The production and safe storage of grains not only affect human food supply but also animal food production because a large portion of grains is used as animal feed. In most places, crops are grown seasonally, and grains are stored for short or long periods after harvesting to meet the needs of food supply chains and to save seeds for the next season. Several studies have reported that maximum losses happen during this operation [1]. It was estimated that the losses of maize grains could be as high as 59.48% after storing them for 90 days in traditional storage structures (Granary/Polypropylene bags) in Sub-Saharan Africa [2]. However, the Food and Agricultural Organization of the United Nations estimated that the total postharvest food losses in 2016, 2020, and 2021 were only 13%, 13.3%, and 13.2%, respectively (FAO, 2021) [3]. This is significantly lower than that reported in some individual countries.

### 1.2. Causes of Postharvest Loss of Cereal Grains

**Figure 1:**
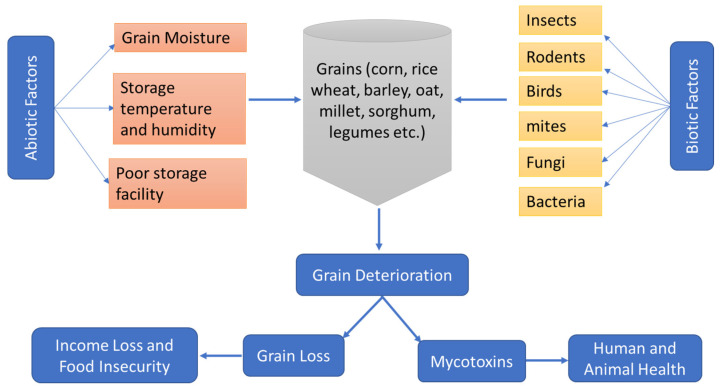
Abiotic and biotic factors causing grain storage loss.

The storage losses are affected by several factors, which can be classified into two main categories: biotic factors and abiotic factors (Figure 1). The abiotic factors are non-living environmental conditions, including grain moisture, storage temperature, and humidity, as well as poor storage facilities [4]. The grain loss caused by abiotic factors can be reduced by sufficient drying and cooling of grains before storage, improving aeration, and reducing air moisture, which is costly for small farmers in developing countries. The biotic factors include insects, pests, rodents, bacteria, and fungi, while the abiotic factors include temperature, humidity, and rain. The damaging effects of biotic factors are influenced by abiotic factors, while the damaging effects of abiotic factors are enhanced by the biotic factors. Among all the biotic factors, insects and pests are considered the most important and cause huge grain losses during storage (30–40%) [4].

#### 1.2.1. Crop Loss Caused by Insect Infestation During Storage

Grain storage insects include grain weevils, grain borers, grain moths, flour moths, grain and flour beetles, mealworms, dermestid beetles, spider beetles, miscellaneous beetles, booklice or psocids, silverfish, cockroaches, flour or grain mites, and parasites of grain pests. Among these insects, the five primary insect pests that cause most of the insect damage to grains in storage and shipment are granary weevil (*Sitophilus granaries*), rice weevil (*Sitophilus oryzae*), maize weevil (*Sitophilus zeamais*), lesser grain borer (*Rhyzopertha dominica*), and Angoumois grain moth (*Sitotroga cerealella*), which is a grain moth that destroys wheat and corn [5]. Generally, the larvae or grubs of the primary insect pests live entirely within the kernel, where they feed unseen and usually unsuspected. They cannot be removed by ordinary cleaning machinery and must be controlled by other means [5]. Stored grain insects cause significant crop losses during postharvest storage. The losses are caused by direct feeding damage or deterioration and contamination of grains.

The storage loss caused by insect infestation of grains was estimated to be 1% to 5% in developed countries and 20% to 50% in developing countries [6]. The insect- and pest-damaged grains are more susceptible to microbial infection. A survey was conducted for three years, from the 2018 to 2020 cropping seasons, to identify storage insect pests and to assess postharvest loss of maize in four major producing areas in Ethiopia. A total of 280 farmers’ fields were surveyed, and *Sitophilus zeamais* was found in most of the farms, with mean grain damage ranging from 6.03% to 31.84% [7]. Insect infestation reduces the quality of stored cereals and legume grains because it causes discoloration, reduces the quality of proteins, carbohydrates, and lipids, and changes the technological characteristics of the raw materials [8].

#### 1.2.2. Crop Loss Caused by Molds During Storage

Due to the relatively low moisture content of stored grains, most microbial infections are caused by molds, not bacteria. Thus, fungi are a major cause of postharvest deterioration of cereals, legumes, and oil seeds [9]. Crop loss caused by molds during storage can be significant, with estimates suggesting that around a third of all produced food is lost due to mold growth, particularly in grains, leading to substantial economic losses and potential health risks from toxins produced by certain molds like aflatoxins. Most grain mold pathogens attach to panicle seeds in the field and grow within the colonized seed, spreading to adjacent seeds during drying, threshing, transport, and storage [10]. Three major mold genera, *Aspergillus*, *Penicillium*, and *Fusarium*, are of importance in food safety because of their ability to produce mycotoxins; however, several other mold genera like *Claviceps*, *Epichloe* and *Neotyphodium*, *Stachybotrys*, *Byssochlamys*, and *Eupenicillum* have recently been identified as mycotoxigenic [11]. The adverse impacts of molds on cereal grains include (1) degrading/reducing grain quality, including decreased nutritional value, discoloration, and odor development; (2) losing seed mass, grain density, seed germination, storage quality, feed efficiency, and grain processing characteristics [12]; and (3) forming mycotoxins, which are hazardous to human, livestock animals, and pets [13].

The toxigenic fungi/molds not only cause quality deterioration and grain loss, but also produce toxic secondary metabolites, mycotoxins, which can cause acute toxicity, death, and chronic diseases such as cancer, immunity suppression, growth impairment, and neural tube defects in humans, livestock animals, and pets. In the stored cereal grains, the major mycotoxin-producing molds are *Aspergillus*, *Fusarium*, and *Penicillium* genera. The mycotoxins important to the safety and economy of cereal grains are aflatoxins (AFs), ochratoxin A (OTA), zearalenone (ZEN), fumonisins, and trichothecenes (including deoxynivalenol (DON), T-2 and H-T2 toxins) [14,15]. Among these mycotoxins, aflatoxin B1 (AFB1) is the most toxic and is responsible for most of the reported mycotoxicoses outbreaks in humans and pets. DON and fumonisins are common in animal feeds, causing reduced performance and different diseases in farm animals, while OTA is frequently detected in infant formula at relatively higher levels [13].

Mycotoxins have significant economic impacts on numerous crops, especially wheat, maize, peanuts and other nut crops, cottonseed, and coffee. Once mycotoxins are produced, they cannot be completely removed through food and feed processing. If the mycotoxin level of the grains is higher than the maximum allowance set by the regulatory agencies, the grains cannot be consumed. In developed countries, the mycotoxin contamination of various crops remains a challenging issue [15,16] because it is considered unavoidable and unpredictable, even when good agricultural, storage, and processing practices are implemented. In the United States, aflatoxin is perennially a problem in corn, peanuts, and cotton seeds [17]. One report estimated that aflatoxin contamination could cause losses to the corn industry, ranging from $52.1 million to $1.68 billion annually in the United States [18].

### 1.3. Problems of Using Pesticides

It is clear that the control of insects, molds, and mycotoxins contamination during storage is extremely important to protect human and domestic animals from mycotoxicoses and to reduce economic losses because cereals are often stored for months in storage bins/silo to meet the demand of supply chain. Conventionally, the insect and mold controls of stored grains are achieved via the sanitation of storage facilities, fumigation using the United States Environmental Protection Agency (EPA) approved pesticides and fungicides, maintaining storage temperature below 4 °C (which is very expensive), and maintaining low moisture in the storage bin [19].

While synthetic pesticides and fungicides have been widely used to control insects and molds in stored grains, their uses present significant environmental and health problems [20]. The environmental impacts include biodiversity reduction, surface and groundwater pollution, soil contamination, and a decrease in fertility. The health problems include direct harmful impacts on humans and other non-target species, increasing resistance against pesticides and residual in the final product. Approximately 3 million cases of acute pesticide poisoning are reported worldwide every year due to the general use of pesticides, including agricultural production and food storage [21]. Types of chronic illnesses associated with pesticide exposure are cancer, Parkinson’s disease, birth defects, asthma, and Alzheimer’s disease. Agriculture workers are at a higher risk of becoming affected with chronic illness due to long-term exposure to pesticides [21].

In addition to preventive pest and mold management practices, such as sanitation, control of moisture and storage temperature as mentioned above, fumigation using synthetic pesticides and fungicides remains an important method. However, these synthetic chemicals are hazardous to the environment and human health, and most of them cannot be used for organic grains. In recent years, the need to develop plant-based food preservatives as an alternative to synthetic chemicals to control losses of food items has become a priority of scientists worldwide, particularly in developing countries [22]. With the increasing demand for organic cereal grains, the need to develop plant-based safer food preservatives has become a priority of scientists worldwide, particularly in developing countries, to protect organic grains from insect infestation, mold contamination, and mycotoxin formation during storage due to the prohibition of synthetic pesticides and fungicides [22]. Essential oils (EOs) have displayed great potential to serve as alternatives to synthetic pesticides and fungicides, but research on how they can be effectively used in grain storage settings is limited. Therefore, the aims of this review are to provide insights into the plant sources of EOs commonly used in the food industry, the chemical compositions of EOs with strong anti-insect and antimicrobial activities, their influencing factors, the relationship between chemical compositions of Eos, their anti-insects and antimicrobial potentials, and current approaches of using EOs for food preservation. It is hoped that this review can promote research on the development of feasible and affordable methodology to apply effective EOs or encapsulated EOs to protect stored grain, particularly, for organic grain protection.

## 2. Essential Oils

### 2.1. Resources of Essential Oils

**Figure 2:**
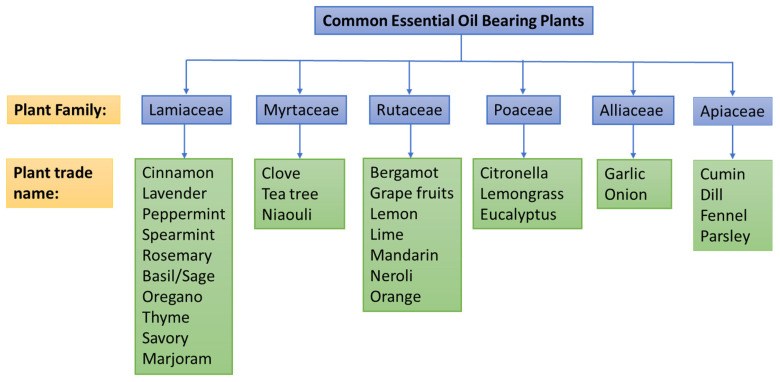
Plant sources of essential oils (EOs) commonly used in the food industry.

Essential oils are the secondary metabolites of aromatic plants, which are volatile, complex molecules with a strong odor. They are defined as “odorous product, usually of complex composition, obtained from a botanically defined plant raw material by steam distillation, dry distillation, or a suitable mechanical process without heating” [23]. Steam distillation is the most commonly used method for essential oil production. An essential oil is a concentrated hydrophobic liquid containing volatile chemical compounds such as terpenes (monoterpenes and sesquerpenes), terpenoids—the oxygenated terpenes (including phenols alcohols, aldehydes, ketones, ethers, esters, oxides, and lactones), benzene derivatives, and miscellaneous compounds and is known to be volatile oils from plants, thus presenting fumigant toxicity [24,25]. Plant materials, such as leaves, flowers, peels, barks, stems, buds, and seeds, can be used to extract volatile odoriferous essential oils. It is estimated that around 3000 essential oils have been isolated from various plant species, but only about 300 of these are commercially valuable or are employed in the cosmetic and food industries [26,27]. The important essential oil-bearing plants include citrus, cinnamon, clove, jasmine, lemongrass, lavender, oregano, mint, thyme, rosemary, and tea trees. Their common names, botanical names, and the families to which they belong can be found in reference [28]. Some widely used EO bearing plants for food industry are shown in Figure 2.

### 2.2. Chemical Compositions of Essential Oils Used in Food Systems

The essential oils (EOs) extracted from citrus peels, cinnamon bark/leaf, clove buds/leaves, jasmine flowers, lemongrass, lavender, oregano flowers, peppermint, thyme, rosemary, and tea trees are commonly used in the food industry. These plants are widely used as spices or herbal medicines and are considered safe for the intended use at the recommended dosage. Thus, the EOs used in the food industry are considered General Recognized as Safe (GRAS). 

**Citrus Essential Oils**: The peels of citrus fruits (*Rutaceae* family), including oranges, lemons, and grapefruits, are rich in essential oil. The chemical composition of citrus essential oils from different types of citrus fruits can be quite different but the dominating component is limonene for all [29]. The concentrations of limonene and other compounds differ with the citrus varieties. Overall, the concentration of limonene is in the order of orange > mandarin (90.7–92.6%) > grapefruit (82.9–92.5%) > navel orange (71.06–74.6%) > lemon > citron (62.8–65.2%) ≥ bergamot > finger citron (32.08–52.44%) ≥ lime [28]. The other main components of citrus EOs were determined to be *α*-pinene, sabinene, *β*-pinene, *β*-myrcene, linalool, linalyl acetate, *γ*-terpinene, *m*-cymene, and 4-terpineol in addition to d-limonene, but not all citrus essential oils contain these components [30]. For instance, a study illustrated that the d-limonene amount was highly dominant (66–93%) in all citrus essential oils and the d-limonene amount of Moro blood orange oil was observed to be higher (93.32%) than in other cultivars. On the other hand, Mayer lemon EO contains 75.50% d-limonene, while Interdonat lemon EO has the lowest amount of d-limonene with 66.58%. The limonene can also be found in the oils of many different plants, including juniper, rosemary, and peppermint [31]. For more complete information regarding the volatile chemical profiles of EOs from different citrus fruits and the factors influencing the chemical makeup, please see the comprehensive review [29].

**Cinnamon Essential Oils**: Chemically, cinnamon oil consists of cinnamaldehyde, linalool, methyl eugenol, limonene, *α*-terpineol, and terpinen-4-ol as primary components [32]. Cinnamon essential oils can be extracted from cinnamon barks, leaves, roots, and fruits of cinnamon tree; they are a plant from the Lauraceae family, but their chemical compositions are very different [33]. The essential oils extracted from bark and leaves of the same cinnamon variety are also different, with bark oil containing highest cinnamaldehyde and leaf oil dominated by eugenol [34]. Bark oil contains about 65–80% of cinnamaldehyde and 5–10% of eugenol, while cinnamon leaf oil constitutes 70–95% of eugenol and 1–5% of cinnamaldehyde. Other abundant constituents are the cinnamyl group, such as cinnamic acid and cinnamyl acetate, compounds containing the endocyclic double bond as *α*-thujene, *α*-terpineol, *α*-cubebene, unconjugated exocyclic double bond eugenol, *β*-caryophyllene, terpinolene, and hydroxyl-substituted aliphatic compounds, such as E-nerolidol, L-borneol, and borneol [35]. The chemical composition of the EOs extracted from the same part of the cinnamon plant varies with the cultivar [35]. A study found the essential oil concentrations and chemical profiles of essential oils extracted from the leaves of five cinnamon varieties are significantly different, and the highest essential oil yield (1.54%) was obtained from *Cinnamomum cassia* leaves followed by *C. zeylanicum*, *C. pauciflorum*, *C. burmannii*, and *C. tamala* [36]. Commercially available cinnamon EOs are mostly extracted from the bark and leaves of cinnamon trees, specifically from species like *C. verum* (also known as *C. zeylanicum*) and *C. cassia*.

**Clove Essential Oils**: Clove oil is extracted from the plant *Eugenia caryophyllus* that belongs to the Myrtaceae family. The clove oil displays insecticidal activity against *Pediculus capitis*, *Anopheles dirus* mosquitoes, and some stored product insects, and suppresses the progeny development of *Tribolium castaneum* and *Sitophilus zeamais*, with isoeugenol being particularly active [37]. The chemical composition of clove oil consists mainly of eugenol oil, which makes up about 78% of the content, and *β*-caryophyllene, which makes up 13%. Another is eugenol, a terpenoid substance that is a member of the phenylpropanoids family. Eugenol is well recognized for its potent, spicy, and aromatic scent, and is present in a variety of plants, primarily in the buds and leaves of the clove plant (*Syzygium aromaticum*), as well as in lesser amounts in plants like cinnamon, basil, and nutmeg [37]. Eugenol has been the subject of extensive research due to its numerous biological activities and potential therapeutic applications.

**Oregano oil**: The major components of the *Oreganum compactum* essential oil are carvacrol and thymol. The chemical composition of oregano essential oils extracted from different species of the oregano family and the same species grown in different countries are summarized in a review [38], which shows that some species have higher carvacrol contents, some are rich in thyme, and a few are dominated by *p*-cymene and terpinene. Linalol (68%) is found in the *Coriandrum sativum* essential oil; *α*- and *β*-thuyone (57%) and camphor (24%) are found in the *Artemisia herba-alba* essential oil; 1,8-cineole (50%) is found in the *Cinnamomum camphora* essential oil [39].

**Rosemary oil**: Rosemary (*Rosmarinus officinalis* L.) is a shrub belonging to the Lamiaceae family, and native to the Mediterranean basin. Rosemary leaf has been widely used in traditional medicine and as a spice in cooking and food processing for its flavor. Rosemary essential oil contains mainly monoterpenes and monoterpene derivatives (95–98%), the remainder (2–5%) being sesquiterpenes [40]. The volatile compounds in rosemary EOs vary widely with grown location, with *p*-cymene, camphor, linalool, *γ*-terpinene, 1,8-cineole borneol, verbenone, *α*-pinene, eucalyptol, camphene, and thymol being the primary components [40,41].

**Thyme oil**: Thyme oil is the hydrophobic extract from *Thymus vulgaris* L. (*T. vulgaris*), a significant aromatic plant with around 100 species in the world; it is a member of the Lamiaceae family. The thyme essential oils can be classified into different chemotypes based on the dominating chemical compounds [42]. Major volatile compounds in thyme essential oil are thymol (21.38–60.15%), *p*-cymene (7.76–43.75%), *γ*-terpinene (4.20–27.62%), carvacrol (1.15–3.04%), and *β*-caryophyllene (1.30–3.07%). Although the concentrations of these compounds vary based on growth region, cultivation practices, and harvest season, the thymol level is the highest, while the carvacrol level is the lowest in thyme oil [43]. These monoterpenic phenols in thyme essential oil possess multiple bioactivities, including strong antibacterial, antifungal, antitumor, anti-inflammatory, antiviral, and antioxidant properties; thus, thyme EOs have been used as medicine and an antimicrobial agent in foods, cosmetics, and toiletries [44].

**Mint oils**: The mint oils are extracted from *Mentha* species which belong to the family Lamiaceae and grow globally. The most economically important mint species are *Mentha aquatica* L., *Mentha canadensis* L., *Mentha spicata* L. (spearmint), and their hybrids *Mentha × piperita* L. (peppermint) [45]. Peppermint essential oil is typically obtained from freshly grounded peppermint flowers or leaves by steam distillation, and its primary components are menthol, menthone, menthofuran, 1,8-cineole, and limonene, whereas, the major components of spearmint include carvone, limonene, *cis*-dihydrocarvone, *trans*-carveol, (E)-*β*-caryophyllene, and germacrene D [46,47]. The phytochemical compositions of essential oils produced from different species of mint grown in different countries can be found in a recent review [44]. The peppermint and spearmint EOs have been highly evidenced for their anti-inflammatory, antibacterial, antiviral, scolicidal, immunomodulatory, antitumor, neuroprotective, antifatigue, and antioxidant activities; thus they are widely used [48,49].

**Tea tree oil**: Tea tree essential oil is extracted from the leaves and branches of the *Melaleuca alternifolia* tea tree, which is a member of the Myrtaceae family. The yield of oil by steam distillation is typically 1 to 2% of the wet plant weight. The primarily volatile compounds in tea tree essential oil are monoterpenes and sesquiterpenes, with the main active ingredients being terpinen-4-ol, *γ*-terpinene, *α*-terpinene, and terpinolene, followed by 1,8-cineole, *α*-pinene, *ρ*-cymene, *α*-terpineol, aromadendrene, *δ*-cadinene, and limonene. The exact chemical composition can vary depending on the source of the tea tree, harvest season, and extraction methods [50,51]. It was reported that tea processing, such as withering, rolling, and fermentation, generally increases the essential oil content, while the drying stage often leads to a decrease in essential oil due to volatile compound loss; this directly impacts the final aroma and flavor profile of the tea significantly [52].

## 3. Factors Influencing the Chemical Compositions of Essential Oils

**Figure 3:**
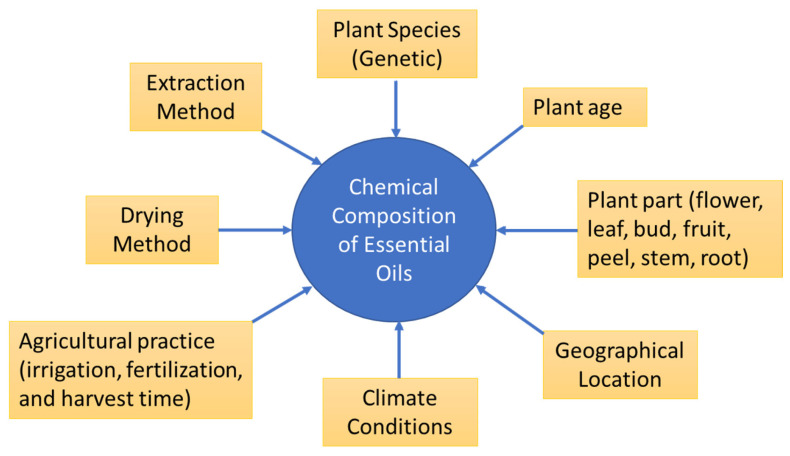
Factors influencing the chemical composition of essential oils.

Each essential oil is a mixture of terpenes (including monoterpenes, sesquiterpenes, and their oxygenated derivatives such as alcohols and aldehydes), as well as phenolic and phenylpropanoid compounds, which are both derived from the acetate mevalonic acid and shikimic acid pathways, respectively [53]. Factors affecting EO yield and composition include plant genetics, growth stage, geographical and environmental conditions (like climate, altitude, and soil type), agricultural methods and practices (plant density, water availability, and harvesting time), plant part used, extraction method, and storage condition [42] as shown in Figure 3. The composition changes significantly influence the biological activity of the essential oil. The chemical compositions of essential oils extracted by steam distillation or hydrodistillation from different plant materials that are grown in various countries are given in Table 1.

### 3.1. Plant Species

The chemical composition of EO is determined by the genotype (species/genera and cultivars), the age/maturity, and the part of the plant [34,54,59]. The chemical profile is influenced by environmental conditions, such as geographical location, climate conditions (temperature, light intensity, and water availability), postharvest drying, and storage conditions, as well as extraction methods [52]. The essential oil composition is governed by biosynthetic pathways, which are under enzymatic control and are genetically determined [71]. Therefore, the concentrations and chemical compositions of EOs originated from different plant species/families or different cultivars of the same species vary greatly (Table 1). A study investigated the chemical compositions of EOs extracted by steam distillation from 14 citrus varieties in China and found that the yield and chemical composition of citrus EOs showed great variabilities. The yield of EOs ranged from 0.95% to 2.8%. D-limonene (39.77–80.13%), *α*-pinene (1.83–13.97%), myrcene (0.8–7.63%), ocimene (0.01–4.52%), and linalool (0.13–8.52%) were found in all samples, *α*-thujene was only found in the EOs of *Changshan huyou* B. Chang, while *ß*-pinene was not found in the EOs from Citrus *limon Burm* F., *Citrus sinensis* Osbeck and *Citrus Reticulate* Blanco Cv. Shatangju [72].

### 3.2. Age/Maturity of Plant

The concentration and composition of essential oils can change throughout the plant’s lifecycle, with the peak often occurring at the flowering stage [73]. One study reported that the essential oil obtained from a two-year-old *Thymus vulgaris* contained more thymol than the one obtained from a five-year-old plant in the same vegetative cycle [74]. Another study found that the essential oil yields of coriander (*Coriandrum sativum* L.) markedly increased during the maturation process. The primary components were geranyl acetate (46.27%), linalool (10.96%), nerol (1.53%), and neral (1.42%) at the immature stage (immature fruits). At the middle stage, linalool (76.33%), cis-dihydrocarvone (3.21%), and geranyl acetate (2.85%) were the main constituents. At the final stage of maturity (mature fruits), the essential oils consist mainly of linalool (87.54%) and cis-dihydrocarvone (2.36%) [75]. *Cinnamon loureirii* bark at 12–15 years old had high EO yields (4.52–5.48%). The major components in the EOs were trans-cinnamaldehyde (50.2–92.9%) and *α*-copaene (0.5–21.3%). The highest content of trans-cinnamaldehyde in bark EOs was obtained from 10- to 12-year-old trees, while the highest content of *α*-copaene was obtained at 13–15 years [76].

### 3.3. Parts of the Plant

Different parts of the plant, like leaves, flowers, stems, or fruits, may yield essential oils with different chemical profiles. As mentioned above, the essential oil from cinnamon bark contains about 65–80% of cinnamaldehyde and 5–10% eugenol, while cinnamon leaf oil constitutes 70–95% of eugenol and 1–5% of cinnamaldehyde [35]. Similar results were reported for other EO-bearing plants (Table 1). A study conducted in China found significant differences in the chemical compositions and antioxidant activities of EOs extracted from three parts of oregano (leaf, stem, and root) [59]. A total of 37 compounds were identified in the leaf–flower oils, with carvacrol (30.73%), thymol (18.81%), *p*-cymene (10.88%), caryophyllene (7.73%), and 3-carene (4.06%) being the major components, which accounted for 72.21% of the total EO composition. Only 11 compounds were identified in the stem oils, with large quantities of palmitic acid (60.18%), linoleic acid (14.25%), carvacrol (6.02%), thymol (3.46%), and oleic acid (5.65%), which accounted for a total of 89.56%. Finally, 29 compounds were identified in the root oils, including large quantities of palmitic acid (58.23%), linoleic acid (12.11%), linolenic acid (3.66%), carvacrol (3.27%), and thymol (1.08%) [54]. The clove bud oil generally has higher concentrations of eugenol and eugenyl acetate, while clove leaf oil contains a higher percentage of sesquiterpenes like *β*-caryophyllene and *α*-humulene, making it slightly milder in scent and potency compared to bud oil [77]

### 3.4. Geographical Locations

Different geographical locations usually differ in climate, altitude, latitude, soil types, and light intensity, which have been reported to influence the chemical compounds of essential oils of similar plant species. As shown in Table 1, rosemary essential oils extracted from *Rosmarius officinalis* grown in Spain, France, Tunisia, and Turkey are different in the number of major compounds and their relative concentrations [40,67,68]. A study evaluated the chemical composition of two different thyme (*Thymus vulgaris* L.) essential oils obtained from France and two from Serbia. The thyme oil from Nyons, France was found to be the linalool chemotype (linalool, 76.2%; linalyl acetate, 14.3%); the oil sample from Jablanicki, Serbia was the geraniol chemotype (geraniol, 59.8%; geranyl acetate, 16.7%); the sample from Pomoravje District, Serbia was of the sabinene hydrate chemotype (cis-sabinene hydrate, 30.8%; trans-sabinene hydrate, 5.0%); and the essential oil from Richerenches, France was of the thymol chemotype (thymol, 47.1%; *p*-cymene, 20.1% [43]. 

### 3.5. Plant Drying Method

The methods used to dry plant materials greatly affect the EO yield and composition. The essential oil underwent different chemical transformation in its monoterpenoids when the same plant material are dried by different methods. For example, a study found that Pulegone (35.0%), menthone (31.1%), and 1,8-cineole (13.0%) were the most abundant in the fresh leaf; the most prominent components in both the air-dried and sun-dried *Mentha longifolia* leaf oils were menthone (47.9% and 38.3%, respectively), while the major compound of oven-dried leaf oil was limonene (40.8%). In addition, oven-drying destroyed all menthone and pulegone, but increased limonene, *α*-Pinene, and *β*-Caryophyllene, in the leaf oil [78]. Another study reported that both convective (CD) and vacuum-microwave (VMD) drying caused a big loss of all major components of rosemary EO, but the combination of these two methods significantly reduced the loss [79]. The drying treatments could significantly change the chemical profiles of essential oils from the leaves where some of the essential oil compounds have been lost and/or increased due to the formation of new constituents by oxidation, glycoside hydrolysis, esterification, and other processes [80].

### 3.6. Essential Oil Extraction Methods

**Figure 4:**
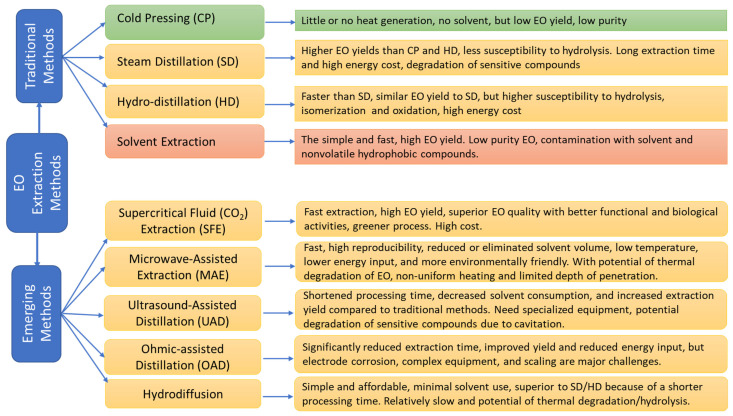
Traditional and emerging methods for the extraction of essential oils [81,82,83,84]. Green color-no organic solvent, no heating, Orange color-no organic solvent but need heating; Red-with organic solvent and heating.

The extraction methods also affect the composition of essential oils. The methods for essential extraction are divided into traditional extraction methods, which include cold press, steam distillation, hydrodistillation, and solvent extraction, and emerging extraction methods, including supercritical fluid extraction (SFE) with CO_2_, microwave-assisted extraction methods, and the ultrasound-assisted extraction method [25,81] Each method has its pros and cons. Some methods enrich different compounds in the extracted oils. The conventional and emerging EO extraction methods, as well as their advantages and disadvantages, are summarized in Figure 4. In addition to the methods themselves, the extraction conditions, or parameters such as temperature, time, particle size of the plant materials, pressure in the SFE, ultrasound frequency, and the energy input level of the microwave, also significantly influence the extraction efficiency and EO composition (Table 2). Therefore, the optimization of the extraction parameter is often needed for each plant material. For example, a study found that steam distillation, hydrodistillation, and SFE of clove buds resulted in similar oil yields, but clove oil obtained by steam distillation contained the highest percentage of eugenol (58.2%); the clove oil obtained by hydrodistillation had the lowest percentage of eugenol (48.82%), while clove oil obtained by SFE contained the highest percentage of eugenol acetate (20.32–21.75%). Soxhlet (solvent) extraction resulted in the highest clove oil yield but the lowest content of eugenol plus eugenol acetate. Furthermore, the extracts from the Soxhlet method are brown ointment, indicating low purity and high organic solvent residue. The extraction yield of SFE was about two times as high as that obtained by steam and hydrodistillation. The essential oil with highest content of eugenol and eugenol acetate was obtained by steam distillation method [55].

**Table 2:** Effects of extraction methods on chemical composition of essential oils from different plant materials.

Aromatic Material	Extraction Methods	Extraction Condition	EO Yield (%)	Major Components (%)	References
Clove buds (*E. caryophyllata* Thunb.)	Supercritical CO_2_ extraction	2 h	18.3–22.4	Eugenol: 53.69 (30 °C)–58.77 (40 °C)	[55]
	Steam distillation (SD)	8–10 h	10.1	Eugenol: 61.2 ± 10.2	
	Hydro-distillation (HD)	4–6 h	11.5	Eugenol: 50.3 ± 3.2	
	Solvent extraction (Soxlet)	6 h	41.8	Eugenol: 30.8 ± 9.3	
Fresh aerial parts of thyme (*T. vulgaris* L.)	Hydro-distillation (HD)	4 h	2.39 ± 0.06	Thymol: 37.20 ± 2.86 *p*-cymene: 16.85 ± 0.08 γ-Terpinene: 8.54 ± 0.02 Carvacrol: 6.81 ± 0.05	[85]
	Microwave assisted HD	2 h	2.52 ± 0.00	Thymol: 40.20 ± 3.03 *p*-cymene: 17.57 ± 0.78 γ-Terpinene: 9.06 ± 0.12 Carvacrol: 6.84 ± 0.68	
Rosemary (*Rosmarinus officinalis* L.)	Hydro-distillation (HD)	3 h	1.35 ± 0.04	Monoterpene: 37.19 *α*-Pinene: 15.82 Camphene: 9.77 *p*-Cymene: 4.79 *β*-Pinene: 3.56 Monoterpenoids: 61.76 Cineole: 31.2 Camphor: 16.5 *α*-Terpineol: 7.1 *β*-Myrcene: 3.75	[41]
	Microwave assisted HD	20 min	1.35 ± 0.04	Monoterpene: 37.19 *α*-Pinene: 15.4 Camphene: 9.16 *p*-Cymene: 4.15 *β*-Pinene: 3.72 Monoterpenoids: 63.64 Cineole: 32.2 Camphor: 16.2 *α*-Terpineol: 7.36 *β*-myrcene: 4.0	
Bakhtiari savory (*Saturejabachtiarica* Bunge.)	Hydro-disillation (HD)	4 h	1.4 ± 0.00	Monoterpenes: 32.86 ± 4.17 Monoterpenoids: 58.36 ± 5.17 Thymol: 28.61 ± 2.99 Carvacrol: 24.98 ± 1.07 Sesquiterpenes: 3.063 ± 0.44	[86]
	Steam distillation (SD)	4 h	1.28 ± 0.03	Monoterpenes: 33.19 ± 4.97 Monoterpenoids: 59.31 ± 3.63 Thymol: 26.81 ± 2.07 Carvacrol: 24.20 ± 0.61 Sesquiterpenes: 5.44 ± 0.32	
	Microwave assisted HD (MHD)	400 W, 40 min	1.03 ± 0.03	Monoterpenes: 28.20 ± 0.27 Monoterpenoids: 65.05 ± 1.6 Thymol: 30.26 ± 0.13 Carvacrol: 26.89 ± 0.95 Sesquiterpenes: 2.96 ± 0.35	
	Microwave assisted steam DH (MSHD)	400 W, 40 min	1.37 ± 0.06	Monoterpenes: 26.231 ± 1 Monoterpenoids: 67.74 ± 1.8 Thymol: 31.190 ± 0.53 Carvacrol: 27.300 ± 0.52 Sesquiterpenes: 2.66 ± 0.03	
Aerial parts of peppermint (*Mentha piperita*)	OAHD	220 V, 50 Hz, 19.71 ± 2.58 min	2.29 ± 0.08	1,8-Cineole: 8.5 ± 0.4 iso-Menthone: 26.1 ± 0.3 Menthofuran:16.3 ± 0.0 neoiso-Menthol: 28.8 ± 0.3 Pulegone: 5.5 ± 0.1	[87]
	MAHD	500 W 16.50 ± 1.01 min	2.17 ± 0.14	1,8-Cineole: 7.2 ± 0.1 iso-Menthone: 24.7 ± 0.1 Menthofuran: 16.6 ± 0.1 neoiso-Menthol: 31.1 ± 0.1 Pulegone: 4.5 ± 0.0	
	HD	55.88 ± 3.30 min	2.29 ± 0.16	1,8-Cineole: 7.6 ± 0.7 iso-Menthone: 26.4 ± 0.0 Menthofuran: 15.5 ± 0.0 neoiso-Menthol: 30.3 ± 0.1 Pulegone: 5.4 ± 0.0	
Tarragon (*Artemisia dracunculus* L.) leaves	HD (control)	2 h	2.08 ± 0.013	Estragole: 76.68 Limonene: 2.86 (Z)-*β*-Ocimen: 8.74 (E)-*β*-Ocimen: 7.90	[88]
	Ultrasound Assisted HD	30 min, 500 W	2.18 ± 0.03	Estragole: 83.07 Limonene: 2.05 (Z)-*β*-Ocimen: 5.82 (E)-*β*-Ocimen: 5.24	

## 4. Essential Oils for Stored Grain Protection

The essential oils have been used to improve flavor and extend the shelf life of food. Examples of using EOs as flavoring agents in the food industry are (1) peppermint, lemon, and orange oils in desserts, candies, and chocolates; (2) thyme oil, a more herbal oil that is better suited for savory foods like stews and sauces; (3) lavender and bergamot oils are popular in chocolate crafting; and (4) rosemary oil as a natural flavor in yogurt. EOs have been applied as bio-preservatives in all types of foods, including fruits and vegetables, fish products, meat products, milk and dairy products, and bread and baked foods for shelf-life extension, mainly due to their antioxidant and antimicrobial properties [89]. In agricultural production and postharvest storage of cereals and legumes, essential oils have been reported to be potential alternatives to synthetic insecticides, herbicides, and fungicides [90]. In the past two decades, many studies have shown that EOs extracted from different plant materials have insecticidal, herbicidal, bactericidal, and fungicidal effects [91].

### 4.1. Insect Repelling and Insecticidal Activity Against Grain Storage Insects

#### 4.1.1. Volatile Essential Oil Compounds with Insecticidal Activity

**Figure 5:**
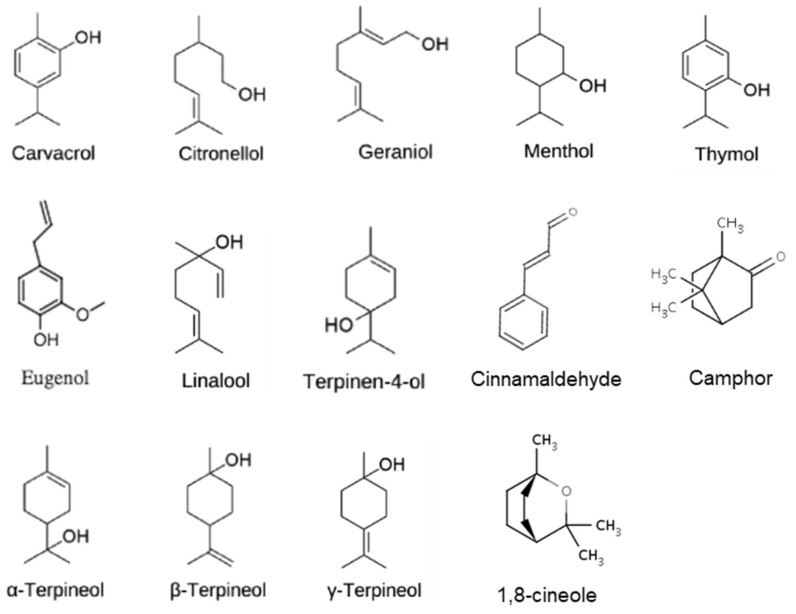
The chemical structures of major compounds in EOs with strong anti-insects and antimicrobial activities.

The insecticidal activity of an EO is determined by the chemical composition of the EO, which is affected by intrinsic and extrinsic factors. The intrinsic factors are related to the plants themselves (species, age, and part), while the extrinsic factors are related to the environmental conditions for plant growth, postharvest handling, and extraction methods [92]. Carvacrol, cinnamaldehyde, citronellal, eugenol, linalool, menthol, 1–8 cineole, and camphor were some of the volatile compounds reported as toxic for some grain storage insects [91]. They are mostly monoterpene alcohols, with the exceptions of cinnamaldehyde and camphor. Their chemical structures are shown in Figure 5. Other chemicals found in EOs with insecticidal properties include *α*-pinene, geraniol, limonene, and methyl jasmonate [93]. For example, thymol was reported to be the most toxic to bed bugs, with an LC_50_ value of 20.50 mg/L, followed by carvacrol (LC_50_  =  46.3 mg/L) and the neurophysiology study results showed that the concentration of 4 mM for both carvacrol (*p*  =  0.005) and thymol (*p*  =  0.001) caused significant neuroinhibition [94]. Eugenol in clove oil or cinnamon leave oil is effective against a wide range of insects, including mosquitoes and flies, while carvacrol, the main compound in oregano and savory EOs, is effective against a wide range of grain storage insects, including the Indian meal moth and Mediterranean flour moth. Menthol in peppermint oil acts as a repellent against mosquitoes, flies, and other insects. Citronellal and citral in lemongrass oil and citrus EOs are repellent and insecticidal properties against mosquitoes and other biting insects. Cinnamaldehyde in the cinnamon bark oil can be used to control insects in stored food products. Linalool, the main constituent of myrtle, linalool is effective against the bean weevil. Other terpenoids, such as 1,8-cineole, eugenol, and menthol, can reduce the number of *Dactylopius. opuntiae* nymphs [95].

#### 4.1.2. Essential Oils Toxic to Grain Storage Insects

Although about 3000 EOs are known to humans and 300 EOs are commercially available, not all EOs are effective insecticidal agents against grain storage insects; some storage insects are resistant to EOs that are toxic to other insects. Table 3 summarizes recent studies on the insecticidal potentials of EOs against grain storage insects. The *Origanum acutidens* essential oil was found to be more toxic against grain storage weevil *Sitophilus granaries*, as compared to its toxicity against *Tribolium confusum*. The *O. acutidens* EO caused 68.3% and 36.7% mortality of *Sitophilus granarius* and *Tribolium confusum* adults, respectively, after 96 h of exposure [96,97]. It was reported that thyme oil significantly reduced the longevity, fertility, and fecundity of chickpea weevils (*Callosobruchus chinensis*) [98]. The thyme oil also significantly suppressed the growth of younger larvae of mealworm but had less effect on the growth of older larvae [99].

A study conducted in Turkey investigated the insecticidal activity of EOs from oregano (*Origanum onites* L.), savory (*Satureja thymbra* L.), and myrtle (*Myrtus communis* L.) against three stored-product insects, including the Mediterranean flour moth *Ephestia kuehniella*, the Indian meal moth *Plodia interpunctella*, and the bean weevil *Acanthoscelides obtectus*. The study found that the insecticidal activity of myrtle oil was more effective against *A. obtectus* adults, with the LC_50_ and LC_99_ values of 33.56 and 50.97 μL/L of air, respectively, while the essential oils of oregano and savory were highly effective against *P. interpunctella* and *E. kuehniella*. The LC_50_ of myrtle, Turkish oregano, and savory EOs were 12.74, 7.52, 10.34 μL/L of air for *E. kuehniella*, and 22.61, 4.06, and 3.43 μL/L of air for *P. interpunctella*, respectively. While the LC_99_ were 29.43, 12.72, and 21.27 μL/L of air for *E. kuehniella*, and 41.74, 5.77, and 7.72 μL/L of air, respectively. Meanwhile, longer exposure times resulted in higher mortality for all tested EOs [99].

A study conducted in Italy evaluated the insecticidal effect of the EOs obtained from eight different plant materials, namely *Mentha longifolia*, *Dysphania ambrosioides*, *Carlina acaulis*, *Trachyspermum ammi*, *Pimpinella anisum*, *Origanum syriacum*, *Cannabis sativa*, and *Hazomalania voyronii*, against two stored-product insect species of economic importance, *Prostephanus runcates* and *Trogoderma granarium*, under a simulated small-scale storage environment [103]. The study found that only *C. acaulis*, *M. longifolia*, and *D. ambrosioides* EOs effectively killed the targeted insects. *C. acaulis* EO at 500 ppm resulted in >97% mortality of *P. truncatus* adults within 3 days on the maize; *M. longifolia* EO at 500 ppm led to 100% mortality of *T. granarium* adults within 2 days on wheat; *D. ambrosioides* EO (500 ppm) killed > 95% of *T. granarium* adults within 4 days on wheat [104].

Demeter and colleagues compared the insecticidal activities of 25 essential oils on the wheat weevil (*Sitophilus granaries*) by using the direct contact method [101]. Each EO was diluted in acetone to concentrations of 1, 2, 3, 4, and 5% (*v*/*v*), respectively; (the Allium sativum EO was diluted to 0.75, 0.5, 0.25, and 0.125%), and 1 mL each of the diluted EOs was applied on 8 g of wheat in 15 mL plastic Falcon tubes. Treated wheat was dried for 15 min under the hood to eliminate the acetone. Then, twenty insects per falcon were added to the wheat and falcon, and were closed by a tulle to allow air circulation. The mortality was recorded after 24 h and 7 days of exposure. Nine out of 25 essential oils led to a mortality of 0 to 60% after 24 h at an EO concentration of 5%, with EOs from *Cinnamomum camphora* CT cinéole being the best at (60%) and EOs from *Citrus limon* and *Myrtus communis* being the least effective at (0%). For most of the tested EOs, the wheat weevil mortality was 0% at a concentration of 1%, except *A. sativum*, which still provoked 75% of mortality after 24 h at that concentration.

A recent study tested the insect repellency and insecticidal activities of five EOs made from cinnamon bark, clove leaves, orange peel, oregano, and thyme against maize weevil (*Sitophilus zeamais*). The repellency of essential oils to the maize weevil was tested using an olfactory meter and the insecticidal activity was tested by using a small lab-scale simulated fumigation method. The cinnamon oil had the highest repellency (90%) of the weevils among the tested EOs. The insecticidal activity test results indicate the mortality of maize weevil increased with EO concentration and storage time. Cinnamon, clove, and thyme oils were more effective than other EOs. The cinnamon EOs and eugenol resulted in 100% maize weevil mortality at week 7 of storage. With the exception of orange essential oils, all others showed great insecticidal activities against corn weevils at a concentration of 12.5 µL/mL, with cinnamon EOs being the most effective against corn weevils [102].

#### 4.1.3. Pesticidal Mechanisms of Essential Oils

The insecticidal activity of essential oil ranges from being lethal to sublethal against a wide range of insects [105]. The mechanisms for essential oils to exert their lethal effect against insects are noted in three ways. First, they act on the nervous system of the insect, and second, they suppress and interfere with normal growth, development, and reproduction. Lastly, they prevent mitochondrial membrane respiratory enzymes or regulate the amount of oxygen that is taken in by the insect, and how much carbon dioxide the insects release [106]. The most important mode of action for essential oil is acting on the nervous system of the insect [107]. Acetylcholinesterase (AChE) molecules in insect nerves, which play a significant role in the maintenance and normal transmission of neural impulses in synaptic clefts, have been the target of many insecticides. For instance, the essential oils from *M. alternifolia* exhibited antifeedant activity and contact toxicity on *Helicoverpa armigera* at a moderate concentration by significantly inhibiting the nerve AChE activity and muscle glutathione S-transferase, an important detoxifying enzyme against pesticides [107]. The inhibitory activity of essential oil against AChE could be attributed to the presence of several active compounds, including terpinen-4-ol, *γ*-terpinene, *α*-terpineol, *α*-terpineol, and 1,8-cineole in the oil [36,108].

### 4.2. Essential Oils with Antifungal Activity for Stored Grain Protection

As aforementioned in Section 1, mold is the primary microorganism responsible for the quality deterioration and safety of stored grains due to the relatively low moisture contents of grains. Many studies have demonstrated that some essential oils (EOs) possess antifungal activities against grain storage fungi. The antifungal effectiveness of EOs and the individual compounds in EOs can be tested using the growth medium agar plating method and expressed at a minimum inhibitory concentration (MIC), the lowest concentration of an EO or a component that prevents the visible growth of bacteria or fungi in a laboratory setting [109].

#### 4.2.1. Chemicals in Essential Oils Responsible for Antifungal Activity

The fungicidal activity of an EO is determined by its chemical composition, and it is influenced by the concentration and exposure time. Not all chemicals in the EO can inhibit the growth of mold cells and block mycotoxin formation. The chemicals in Eos are primarily responsible for their fungicidal activity are phenolic compounds like thymol, carvacrol, eugenol, cinnamaldehyde, and various terpenes, which can disrupt fungal cell membranes due to their lipophilic nature and low molecular weight; these are commonly found in oils like thyme, oregano, tea tree, cinnamon, and clove oil [110].

#### 4.2.2. Essential Oils with Fungicidal Potential Against Grain Storage Molds

Most antifungal studies of natural plant extracts were conducted on growth media; only a limited number of studies have been conducted with grains. Table 4 summarizes some recent studies related to the antifungal activity of some common EOs.

An early study using the fumigation method revealed that oregano EO effectively inhibited the growth of *Asperigillus* species in stored wheat grains and could inactivate the mold spores at a vapor concentration of 0.2 µL/L of air, while thyme EO was less effective [111,112]. The study on the antifungal potential of citrus EO found that 15 min of vapor treatments per day of wheat grain via Citri-VTM^®^ vapor reduced the growth of *A. niger* and *P. chrysogenum* on grain by 50–60% over 10 days [112,113]. The vapors from undiluted mint and oregano EOs were found to completely inhibit the fungi that caused bunt disease on wheat seeds over 7 days, but the vapor from 10% of these EOs showed 0% inhibition [113]. This indicates that bunt disease-causing fungi have strong resistance to mint and oregano EOs, and a high EO concentration is needed to effectively inhibit fungi. It was confirmed by the agar diffusion assay that the EOs from cinnamon, anise, clove, citronella, pepper, peppermint, and camphor could inhibit the growth of three Ochratoxin A (OTA)-producing molds *Aspergillus niger*, *A. oryzae*, and *A. ochraceus*, with cinnamon EO being the most effective of all the fungi strains, followed by the clove EO, while the rest of the tested EOs exhibited moderate antifungal activity. The study also demonstrated the antifungal protection of cinnamon and clove EOs in bread preservation [114]. The cinnamon and clove EOs also inhibited mold growth on green bean cake and finger citron crisp cake in another study [119].

It was reported that the main chemical compounds in tea tree essential oils, such as terpinen-4-ol, *α*-terpineol, linalool, *α*-pinene, and *β*-pinene, displayed the highest fungicidal activity with minimum inhibitory concentrations and minimum fungicidal concentrations of ≤0.25%, followed by 1,8-cineole [120]. The fumigation vapor of tea tree oil completely inhibited the growth of *A. flavus* on agar plates at a concentration of 1.714 μL/mL [115]. However, compared to EOs such as thyme oil and lemongrass oil, the fungicidal activity of tea tree oil is weak [121]. Although tea tree EO displayed strong antibacterial activity in different studies, its antimicrobial activity against yeast species and biofilm-forming bacteria strains was weak [51].

It was reported that the spearmint EO significantly inhibited the growth of *A. flavus* and the formation of aflatoxin B1 at concentrations of 1.0 and 0.9 µL/mL, respectively. The EO also had a broad fungitoxic effect against 19 other storage molds isolated from chickpea and wheat mycoflora, including *Alternaria alternate*, the other six *Aspergillus* strains, *Fusarium oxysporum*, and the four *Penicillium* strains [101]. The essential oil of corn mint (*Mentha arvensis*) was considered best for inhibiting the growth of two DON-producing fungi *F. proliferatum* and *F. verticillioides*, while the oils of thyme (*Thymus vulgaris*) and Dill seed (*Anethum graveolens*) showed high fungicidal efficacy [122]. However, the spearmint (*Mentha spicata* var. crispa) essential oil at a concentration of 0.5 mL/L moderately inhibited the growth of *P. expansum* (inhibition zone of 11.46 ± 0.63 mm) and *P. crustosum* (inhibition zone of 12.93 ± 0.46 mm), but its inhibition on the growth of *P. citrinum* was weak [123]. A study found that thymol, the primary compound in thyme EO, disrupts fungal cell walls and cell membranes by increasing the generation of reactive oxygen species (ROS) on the fungal cell surface and blocking the fungal molecular genes required for cell wall fortification and cell membrane synthesis [124].

Essential oils not only inhibit mold growth but also inhibit the formation of mycotoxins. In the study by Perczak and colleagues, EOs from cinnamon bark (*Cinnamomum zeylanicum*, Indonesia), oregano herb (Origanum vulgare, Mediterranean countries), palmarosa leaves (*Cymbopogon martini*, India), orange peel (*Citrus aurantium* dulcis, Brazil), verbena leaves and flowers (*Thymus hiemalis*, Spain), spearmint leaves (*Mentha viridis*, China), fennel seeds (*Foeniculum vulgare* dulce, Russia/Bulgaria), and rosewood (*Aniba rosaeodora*, India) were screened for their antifungal activities against *Fusarium graminearum* and *F. culmorum*, which are widely present in the stored grains [109]. The cinnamon, oregano, and palmarosa EOs demonstrated the highest antifungal activity, while spearmint, fennel, rosewood, and orange EOs showed similar but much lower efficacy in the inhibition of tested fungi. With the exception of orange peel EO, the treatment of wheat grains with tested EOs reduced the concentrations of ergosterol by 68–90%, zearalenone by 99–100%, and total group B trichothecene (including DON, FUSX, 3-AcDON, and 15-AcDON) by 94.5–100% [109]. Another study examined the fungicidal activities of cinnamon bark, clove, orange peel, oregano, and thyme against the molds isolated from moldy organic corn grains using growth medium agar plates containing different concentrations of EO. The results show that the cinnamon, clove, oregano, and thyme EOs completely inhibited the growth of molds at concentrations of 0.4, 0.6, 0.6, and 0.8 mg/mL, respectively, while the EO from orange peel did not show any inhibitory activity against the corn grain molds. The study also found that Fusarium species were more sensitive to the tested EOs and were completely inhibited at 1/10 of the EO concentration for *Aspergillus* and *Penicillium* species [118]. The simulated fumigation of organic corn grains in a laboratory setting revealed that the cinnamon, clove, oregano, and thyme EOs not only significantly inhibited the growth of mold, but also remarkably reduced the concentrations of aflatoxin B1, B2, G1, and G2 in the organic corn grains during a 6-week storage period at 25 and 35 °C [118].

#### 4.2.3. Antifungal Mechanism of Essential Oils

The antimicrobial properties of essential oils include antibacterial, antiviral, and fungicidal activities. The antimicrobial mechanism of EOs is usually not a single model due to the complex of the EO composition. The antifungal activity of EOs is caused by the terpenes and terpenoids in the oil that can disrupt the cell membrane, cause cell death, or inhibit the sporulation and germination of food spoilage fungi due to their highly lipophilic nature and low molecular weight [125]. The possible mechanisms include (1) disruption of cell wall and cell membrane, thus increasing the permeability of cell membranes. The majority of research has revealed that the cytotoxic nature of EOs and their constituents are related to their capacity to rupture cell walls and cell membranes, coagulate the cytoplasm, and, as a result, cause damage to cellular organelles, and the leakage of macromolecules [126]. (2) Many essential oil components are lipophilic or hydrophobic in nature, and they are able to interact directly with the fungal membrane, which results in a change in the characteristics of the membrane, including the fluidity of the membrane, but there is no evidence of active transport occurring through trans-membrane pump [127,128]. The hydrophobic components in EO can affect cell membrane permeability for cations, like Ca^2+^, H^+^, and K^+^. This changes the flow of protons, altering the chemical composition of cells and their activity [53]. (3) At the molecular level, EOs significantly suppress the expression levels of certain genes related to glycolysis/gluconeogenesis, starch, and sucrose metabolism along with the accumulation of metabolites, leading to energy metabolism disorder and growth stagnation in *F. oxysporum* [129,130]. Some EOs can affect mitochondrial effectiveness by inhibiting the action of mitochondrial dehydrogenases [131].

### 4.3. Antibacterial Activity of Essential Oils

Although bacterial contamination/infection has not been a major concern for stored grains, The bacterial species that occur commonly on grain are generally non-pathogenic, but pathogens such as Salmonella, Escherichia coli, and Bacillus cereus can occur [132]. The bacterial contamination of stored grains may be caused by soil and bird drops during sun drying. The growth of bacteria in stored grains during storage is affected by moisture (relative humidity) and temperature. Thus, in the tropical areas, where the temperature and air moisture are high, bacterial contamination can also cause significant grain loss, and even food safety issues in addition to molds [133]. Generally, Gram-positive bacteria are more susceptible to EOs than Gram-negative bacteria because around 90–95% of the cell wall material of Gram-positive bacteria is peptidoglycan, which allows hydrophobic EO molecules to easily penetrate the cells and act on both the cell wall and within the cytoplasm, while the cell wall of Gram-negative bacteria is more complex. It has a peptidoglycan layer that is thinner than in the cell wall of Gram-positive bacteria, which is covered by an outer membrane composed of a double layer of phospholipids that are linked to the inner membrane by lipopolysaccharides (LPS). The LPS consists of lipid A, the core polysaccharide, and the O-side chain, which provides the “quid” that allows Gram-negative bacteria to be more resistant to EOs and other natural extracts with antimicrobial activity [134]. The previous studies confirmed that most EO terpenes, such as *p*-cymene, limonene, terpinene, sabinene, and pinene, do not possess or only have weak antibacterial activity [135,136]. Instead, the EOs rich in terpenoids, such as thymol, carvacrol, linalool, menthol, geraniol, linalyl acetate, citronellal, and piperitone, often have strong bactericidal activity [137]. The antibacterial potential studies with the disk diffusion method found that tea tree essential oil effectively inhibited the growth of both Gram-positive and Gram-negative bacteria, as well as fungi, such as Enterococcus faecalis, Staphylococcus aureus, and Candida albicans, while yeast species and biofilm-forming bacteria strains were more resistant [50,138]. The methods used to determine the antibacterial activities of EOs include diffusion, disk-diffusion, and broth or agar dilution.

The antibacterial mechanisms of EOs are similar to those of the antifungal mechanism mentioned above. For example, the EO from Chuzhou chrysanthemum could lead to the leakage of proteins of *E. coli* and *S. aureus* cells, resulting in a 47.21% and 36.44% drop in protein content after EO treatments, respectively. The SDS-PAGE results confirmed the disruption of bacterial cytoplasmic membrane by EO. The EO also affected the hexose monophophate pathway of *E. coli* and the embden-meyerhof-parnas pathway of *S. aureus*. Furthermore, the result of the DNA topoisomerase inhibition assay showed that the activity of topoisomerase I and topoisomerase II was inhibited by EO treatment. For E. coli, the hexose monophophate pathway was affected by the decrease in the activity of the key enzyme. For *S. aureus*, the activity of three key enzymes was reduced by inhibiting the embden-meyerhof-parnas pathway [139]. In addition, EOs can inhibit the growth of bacteria by affecting their energy metabolism [134,139].

## 5. Safety Concerns of Using Essential Oils in Food Preservation

Most essential oils (EOs) are considered safe for consumers and have been granted Generally Recognized as Safe (GRAS) status by the FDA in the United States [140]. Popular spices like clove, cinnamon, basil, nutmeg, thyme, and oregano contain valuable EOs. The EOs find applications in several industries, including food and beverage, pharmaceutical, home care, cosmetics, and aromatherapy. Their natural antimicrobial properties, antioxidant potential, pleasant aroma, and flavor make them suitable for use in the food industry [141]. The EO-based pesticides are beginning to reach the marketplace in the European Union, India, and China. However, some compounds, including monoterpenes such as *α*-terpinene, camphor, citral, limonene, pulegone, and thujone in the essential oils exhibited different toxicities in the cell culture and rat studies, with limonene being less toxic [142,143]. Due to the presence of these compounds in most EOs, human beings exposed to high doses of EO can be poisoned [144]. Therefore, The European regulatory authority authorizes only some crude essential oils (clove oil, sweet orange peel oil, lemon grass oil, and mint oil) as active ingredients for the formulation of commercial pesticides, but single constituents are not allowed. Yet, several plant EOs and their constituents are exempt from registration in the United States.

## 6. Methods of Essential Oil Application for Stored Grain Protection

### 6.1. Challenges of Using Essential Oils for Stored Grain Preservation

Although many EOs have shown excellent potential as alternatives to synthetic pesticides and fungicides, applying EOs to grains on a large scale is a major concern. In limited studies, grains were soaked in diluted essential oil solution followed by drying [145]. This is infeasible for large storage facilities, and it may also significantly affect the sensory characteristics of the grains because of the strong odor of the EOs. Simulated fumigation seems to be the preferred method, as it should be more useful and realistic for applications during storage. However, the application of EOs in free form in food is limited due to (a) highly volatile and rapid releases from applied surfaces, and thus frequent reapplications may be needed for long-term storage, which will result in high cost; (b) the possibility to cause undesirable sensory characteristics to the food due to the strong aroma/odor; (c) instability, easy oxidation, and degradation of bioactive compounds in a short period of time; and (d), considerable loss in EO biological activity [146]. These factors can reduce their effectiveness when used for grain protection. Encapsulation is an efficient approach to overcome these drawbacks and keep the original characteristics of EOs.

### 6.2. Use Encapsulation Technology to Improve the Applicability of Essential Oils

Encapsulation is a process that entraps active agents within a carrier material. Some of the most used carriers are natural polymers, including polysaccharides (alginate, cellulose derivatives, chitosan, starch, and cyclodextrin), proteins (e.g., soy protein, zein, gelatin, and whey protein), and lipids, such as wax [147,148]. These encapsulants must be biodegradable and safe for human health. Among these natural polymers, chitosan and *β*-cyclodetrin (*β*-CD) have been reported to have a low production cost, good biodegradability, and good biocompatibility, with no adverse effects on health and the environment. Chitosan is the most studied and the most appropriate nanocarrier for the delivery of EOs because of its abundance, its GRAS status, its high encapsulation efficiency, and its controlled release of EOs [149]. Over the past two decades, microencapsulation and nanoencapsulation technologies have been developed to solve some of the problems related to the applications of EOs in the agricultural and food industries. The encapsulation can control the release rate of volatile compounds in EOs and reduce the impacts of EO odor on the sensory quality of food products due to treatment [149,150,151]. There are a variety of encapsulation techniques, such as coacervation, emulsification, spray drying, complexation, ionic gelation, nanoprecipitation, film hydration, etc. The selection of the encapsulation method depends on the intended end uses [148].

Some studies found that direct applications of nano-encapsulated EO emulsions on fruit surfaces by dipping, spraying, or coating effectively delayed the decay of fruits caused by the fungus during storage [149,152,153]. Encapsulated EOs such as peppermint oil, and EOs rich in carvacrol and linalool, also shower higher fumigant efficacy against *T. castaneum* and *S. oryzae* in stored foods than their free forms [154,155]. A recent study found that the encapsulated *Cinnamon cassia* EOs effectively inhibited the growth of *Penicillium crustosum*, *Alternaria alternata*, and *Aspergillus flavus* in maize flour without causing undesirable aroma issues compared to the free cinnamon EO [156].

## 7. Conclusions and Future Perspectives

This review clearly illustrates the potential of some essential oils to serve as alternatives to synthetic pesticides, fungicides, and bactericides for stored grain preservation. However, these bioactive activities vary with chemical composition and EO concentrations (actually, the concentrations of effective components in the EOs). Because the chemical compositions of EOs from different parts of the same plant are significantly different and are affected by the plant’s age and environmental factors, it is important to standardize the cultivation condition/practice and harvest the plant at an optimal growing stage to obtain EOs with relatively stable compositions for specific applications. In addition, the chemical composition of EO from one plant is affected by the geographical location and climate conditions where the plant grows, which cannot be controlled by humans; thus, the bioactivities of an EO produced in different regions of the world may be quite different and may vary from year to year. Therefore, it is important to check the concentrations of active compounds in the EOs to determine the concentration to be used for a specific purpose. Although numerous studies on the encapsulation of EOs have been published, the application of EO micro- or nano-capsules seems limited to the preservation of fresh produce and some processed food products. Thus, more studies are needed for the development of EO application methods for postharvest grain protection. Additionally, studies in the farm or industrial grain storage setting are needed to assess the feasibility and cost-effectiveness of using EOs or encapsulated EOs as pesticide and fungicide alternatives. Further, the sensory characteristics of food products made from EOs or encapsulated EO-treated grains also need to be investigated because they play a critical role in consumer acceptance.

## Figures and Tables

**Table 1:** Major components of some common essential oils and their plant sources.

Essential Oils	Plant Source	Part of Plant	Country	Major Components and Their Concentration	References
Clove oil	*Syzygium aromaticum* L.	Leaf	Bangladesh	Eugenol (74.28%), eucalyptol (5.78%), caryophyllene (3.85%), *α*-cadinol (2.43%), limonene (2.08%), and *α*caryophyllene (1.52%)	[54]
	*E. caryophyllata* Thunb	Bud	China	Eugenol (48.8–58.2%), caryophyllene (17.5–36.9), eugenol acetate (13.8–20.5), *α*-humulene (2.61–4.41%), *α*-copaene (0.81–1.93%,), and cadinene (0.66–1.42%)	[55]
Clove oil	*Syzygium aromaticum*	Bud	Indonesia	Eugenol (55.60%), eugenyl acetate (20.54%), caryophyllene (14.84%), and *α*-humulene (2.75%).	[56]
Manado clove oil	*Syzygium aromaticum*	Bud	Indonesia	Eugenol (74.64%), caryophyllene (12.79%), eugenyl acetate (8.70%), and *α*-humulene (1.53%)	[56]
Cinnamon oil	*Cinnamomum altissimum* Kosterm	Bark		Cinnamonaldehyde: (65–80%), eugenol (5–10%), linalool (36.0%), methyl eugenol: 12.8% limonene: 8.3%, *α*-terpineol (7.8%), and terpinen-4-ol (6.4%)	[26,32]
	*Cinnamomum zeylanicum* Blume	Bark	India	(e)-cinnamaldehyde (97.7%), *δ*-cadinene (0.9%), *α*-Copaene (0.8%), and *α*-amorphene (0.5%)	[34]
	*Cinnamomum zeylanicum* Blume	Leaf	India	Eugenol (87.3%), bicyclogermacrene (3.6%), *β*-caryophyllene (1.9%), *α*-phellandrene (1.9%), aromadendrene 1.1%, and *p*-cymene 0.7%, 1,8-cineole (0.7%), germacrene-D (0.6%), *α*-pinene (0.5%), and spathulenol (0.5%)	[34]
Oregano oil	*Origanum vulgare* L.	Leaf	Salerno, Italy	Carvacrol (56.2–81.4%), *p*-cymene (1.6–17.7%), *γ*-terpinene (0.8–14.2%), *α*-pinene (0.1–2.1%), thymol methyl ether (0.4–1.8%), and thimoquinone (0.5–3.5%)	[57]
Oregano	*Origanum acutidens*	Flower	Sivas, Turkey	Carvacrol (66.2%), *p*-cymene (9.15%), *β*-caryophylene (4.43%), and γ-terpinene (3.54%)	[58]
Oregano	*Origanum vulgare* L.	Leaf-flower	Hebei, China	Carvacrol (30.73%), thymol (18.81%), *p*-cymene (10.88%), caryophyllene (7.73%), and 3-carene (4.06%)	[59]
Oregano	*Origanum vulgare* L.	Stem and root	Hebei, China	Palmitic acid (60.18–58.56%), linoleic acid (14.25–12.11%), carvacrol (6.02–3.27%), thymol (3.46–1.08%), and oleic acid (5.65%)	[59]
Thyme EO	*Thymus vulgaris* L. (*T. vulgari*)			Thymol (21.38–60.15%), *p*-cymene (7.76–43.75%), *γ*-terpinene (4.20–27.62%), carvacrol (1.15–3.04%), and *β*-caryophyllene (1.30–3.07%)	[26,60,61]
Thyme EO	*Thymus vulgaris* L.		Nyons, France	linalool, 76.2%; linalyl acetate, 14.3%	[43]
			Richerenches, France	thymol, 47.1%; *p*-cymene, 20.1%	
			Jablanicki, Serbia	geraniol, 59.8%; geranyl acetate, 16.7%	
			Pomoravje District, Serbia	cis-sabinene hydrate, 30.8%; trans-sabinene hydrate, 5.0%	
Coriander leave EO	*Coriandrum sativum* L.	Leaves	Bangladesh	2-decenoic acid (30.8%), E-11-tetradecenoic acid (13.4%), capric acid (12.7%), undecyl alcohol (6.4%), tridecanoic acid (5.5%), and undecanoic acid (7.1%)	[62]
Coriander seed EO	*Coriandrum sativum* L.	Seed	Bangladesh	Linalool (37.7%), geranyl acetate (17.6%), and Î^3^-terpinene (14.4%)	[62]
Cilantro oil	*Coriandrum sativum* L.	leaves	Saudi Arabia	1-decanol (17.85%), decanal (11.04%), trans-2-dodecen-1-ol (7.87%), menthone (6.71%), 2-decen-1-ol, trans- (5.44%), dodecanal (4.76%), trans-tetradec-2-enal (3.14%), and sedanolide (3.02), and thymol (3.01%)	[63]
Citrus peel EO	Lemon (*Citrus limon*)	Peel	Turkey	Limonene (61.8%), *β*-pinene (8.1%), γ-terpinene (10.6%), *β*-bisabolene (1.6%), and *β*-caryophyllene	[64]
	*Orange*	Peel	China	D-limonene (91.12%), myrcene (4.11%), *α*-pinene (1.11%), linalool (0.65%), *α*-terpineol (0.60%), decanal (0.44%), sabinene (0.41%), and oxygenated monoterpenes (1.78%)	[65]
	Sour orange (*C. aurantium*)	Peel	Turkey	Limonene (94.1%), *β*-pinene (0.5%), myrcene (1.8%), *β*-caryophyllene (0.1%), geranyl acetate (0.08%), linalyl acetate (1.2%), geranial (0.1%), decanal (0.2%) and linalool (0.4%)	[66]
	Sweet orange (*C. sinensis*)	Peel	Turkey	Limonene (91.6%), myrcene (1.3%), sabinene (1.0%) and *α*-pinene (0.9%) as the major monoterpenes, octanal (1.4%), and linalool (0.4%)	[66]
	Mandarin (*C. reticulata*)	Peel	Turkey	Limonene (90.7%), myrcene (2.1%), γ-terpinene (3.9%), *α*-pinene (0.5%), and sabinene (0.3%),	[66]
Rosemary	*Rosmarius officinalis*	leaf	Spain	*α*-pinene (19.4–24.7%), 1,8-cineole (19.0–21.8%), and camphor (16.3–18.9%)	[67]
Rosemary	*Rosmarius officinalis*	leaf	French	*α*-pinene (19.9–35.1%), 1,8-cineole (5.3–24.8%), and bornyl acetate (1.2–14.3%)	[67]
Rosemary	*Rosmarius officinalis*	leaf	Tunisia	1,8-cineol (33.08–37.75%), camphor (13.55–18.13%), *α*-pinene (8.58–9.32%), *α*-terpineol (6.798.17%), camphene (5.07–5.58%), borneol (4.08–5.48%), limonene (3.19–3.04%), and *p*-cymene (2.42–3.11%)	[68]
Peppermint	*Mentha piperita* L.	flower	Iran	Menthol (36.9%), menthone (28.8%), methyl acetate (4.5%), carveone (3.8%), neomenthol (3.8%), 1,8-cineole (3.8%), limonene (3.29%), menthofuran (1.9%), trans caryophyllene (1.61%), *β*-cubebene (1.3%), and trans-anethole (1.26%)	[69]
Peppermint	*Mentha piperita* L.	Leaf (fresh)	Iran	Menthol (44.39%), menthone (15.36%), menthofuran (10.27%), 1,8-cineole (5.81%), menthyl acetate (4.78%), neoisomenthol (2.37%), and limonene (1.87%)	[69]
Peppermint, Native	*Mentha piperita* L.	Leaf	USA	menthol (38.45%), menthone (21.8%), 1,8-cineole (5.62%), and neo-menthol (4.19%), isomenthone (3.75%), menthofuran (2.08%) beta-Caryophyllene (2.87%), germacrene D (3.24%), and limonene (1.58)	[47]
Spearmint	*Mentha spicata* L.	Leaf	USA	carvone (70.36%), limonene (6.96%), 1,8-cineole (2.24%), *β*-bourbonene (1.69%), *β*-caryophyllene (1.1%), germacrene (1.01%), cis-Dihydrocarvone (0.99%)	[47]
Tea tree	*Melaleuca alternifolia*	leaf	Serbia	Terpinen-4-ol (40.3%), γ-terpinene (11.7%), 1,8-cineole (7.0%), and *p*-cymene (6.2%)	[51]
Tea tree	*Melaleuca alternifolia*	leaf	Australia	Terpinen-4-ol (40.1%), γ-terpinene (23.0%), *α*-terpinene (10.4%), 1,8-cineole (5.1%), terpinolene (3.1), *p*-cymene (2.9%), *α*-Pinene (2.6), *α*-terpineol (2.4%), aromadendrene (1.5%), *δ*-Cadinene (1.3%), and limonene (1.0%)	[70]

**Table 3:** Essential oils with insecticidal potential for grain protection during storage.

Essential Oils	Insect Species	EO Concentration	Mortality (%) or Study Results	References
*Origanum acutidens* oil	*Sitophilus granaries*	375 μL/L air	68.3% (96 h)	[97]
	*Tribolium confusum*	375 μL/L air	36.7% (96 h)	
Thyme Oil *Thymus vulgarus*	Chickpea weevil	10 μL/Petri dish	100% (24 h)	[98]
Myrtle oil	bean weevil (*A. obtectus*)	25 μL/L air	100% (24 h)	[99]
Oregano oil	Indian meal moths and Mediterranean flour moths	25 μL/L air	100% (24 h)	
Savory oil	Indian meal moths and Mediterranean flour moths	25 μL/L air	100% (24 h)	
Spearmint (*Mentha spicata* L.) EO	C. chinensis		The oil caused 100% mortality to C. chinensis after 24 h of treatment and 100% repellency at 0.025 µL/mL 1 air. The effective fumigant, showing 98.46% oviposition deterrency, 100% ovicidal activity, 88.84% larvicidal activity, 72.91% pupaecidal activity, and 100% antifeedant activity at 0.1 µL/mL of air.	[100]
25 essential oils from *Allium sativum* (*garlic*), *Gaultheria procumbens*, *Mentha Arvensis*, *Eucalyptus dives*, etc.	*Sitophilus granarius*		*Allium sativum* EO was the most toxic essential oil, with the lowest LC_90_ both after 24 h and 7 days. *Gaultheria procumbens*, *Mentha arvensis*, and *Eucalyptus dives* oils appeared to have a good potential in terms of toxicity/cost ratio. Other EOs were less/not effective.	[101]
Cinnamon EO	Maize Weevil (*Sitophilus zeamais*)	62.5–250 μL/L air	52.9–100%	[102]
Clove EO			44.1–70%	
Origanum EO			30.0–63.3%	
Thyme EO			32.4–93.3%	

**Table 4:** Essential oils with fungicidal activity for grain protection during storage.

Essential Oils	Application Method	Fungi Species	EO Concentration	MIC or Percentage of Inhibition	References
Oregano oil	Fumigation	*Aspergillus flavus*, *Aspergillus niger* and *Aspergillus ochraceus*	2.0 µL/L of air	2.0 µL/L of air to inhibit the mycelial growth of the fungi while spores were eradicated following exposure to 2.0 to 2.5 µL/L.	[111]
Thyme oil	Fumigation	*Aspergillus flavus*, *Aspergillus niger* and *Aspergillus ochraceus*	2.0–2.5 µL/L of air	More than 4.0 µL/L was needed to control mycelia and cell growth following exposure. MIC for mold spores was 3.0 µL/L.	[111]
Citrus EO	Simulated fumigation	*Aspergillus niger* and *Penicillium chrysogenum*		50–60% inhibition over 10 days	[112]
Clove, mint oregano and thyme oils	Blotter method	Wheat borne fungi including a mixture of *Acremonium*, *Alternaria*, *Arhrobotrys*, *Aspergillus*, *Cladosporium*, *Epicoccum*, *Fusarium*, *Penicillium*, *Rhizopus*, *Trichoderma* and *Ulocladium* genera	0.05–10%	Highest doses totally inhibited the fungal growth on wheat seeds, but they also inhibited the germination of seeds. Lower doses failed to inhibit fungi on seeds, but they decreased or totally inhibited the bunt spore germination	[113]
Clove, mint oregano and thyme oils	Fumigation	Wheat borne fungi: a mixture of *Acremonium*, *Alternaria*, *Arhrobotrys*, *Aspergillus*, *Fusarium*, *Penicillium*, *Rhizopus*, *Trichoderma* etc.	10% and undiluted (100%)	10% of these EOs showed 0% inhibition. Undiluted EOs inhibited the fungi completely	[113]
Cinnamon and Clove EOs	Agar diffusion assay	*OTA* producing fungi: *Aspergillus niger*, *A. oryzae*, and *A. ochraceus*	0.16–0.24 mg/mL	MIC of cinnamon EO: 0.0625 to 0.125 mg/mL MIC of clove EO: 0.25 mg/mL	[114]
Spearmint EO		Alternaria alternate, seven *Aspergillus* strains *Aspergillus flavus*, *Fusarium oxysporum*, four *penicillium* strains		The EO significantly inhibited growth and aflatoxin B1 production of Aspergillus flavus at 1.0 and 0.9 µL/mL	[102]
Tea Tree EO	Agar plates	*Aspergillus flavus*	1.714 μL/mL	Completely inhibited the growth of Aspergillus flavus onagar plates at a 1.714 μL/mL.	[115]
Thyme, oregano, lemongrass, clove, and cajeput EOs	Disk volatilization	*Aspergillus* spp., *Fusarium* spp., *Penicillium ochr-ochloron*	250, 125, and 62.5 μL/L of air	Complete inhibition of fungal growth. The efficiency of EOs followed this order: thyme > oregano > lemongrass > clove > cajeput.	[116]
Cinnamon, clove, garlic, peppermint EOs	PDA medium plate	*Aspergillus* and *Penicillium species* isolated from wheat grains	0.0625, 0.125, 0.25, 0.5, 1, 2 and 4% *v*/*w* (EO in tween 20)	At EO concentration 0.12%, cinnamon EO completely inhibited fungal growth and suppressed aflatoxins production other EOs exhibited about 50% reduction on the mycelial.	[117]
Cinnamon, Origanum, Thyme and Clove EOs	Growth medium plates	All molds from corn moldy grains	0.05–0.8 mg/mL	IC_50_: 0.05 (cinnamon)–0.28 (thyme)	[118]
	Growth medium plates	*Fusarium species*	0.001–0.05 mg/mL	IC_50_: 0.006 (cinnamon)–0.04 (thyme)	
	Simulated fumigation	All molds from corn moldy grains	1.25 µL/mL of air	100% inhibition over 6 weeks	

## Data Availability

The data presented in this article are publicly available in the literatures cited in this manuscript.
